# Balancing Ubiquitination and Deubiquitination in Apoptotic Protein Stability and Cancer Cell Survival

**DOI:** 10.3390/ijms27188049

**Published:** 2026-09-10

**Authors:** Miso Choi, Kwang-Hyun Baek

**Affiliations:** Department of Biomedical Science, CHA University, 335 Pangyo-ro, Bundang-gu, Seongnam-si 13488, Republic of Korea; cbo123456@naver.com

**Keywords:** apoptosome, cancer, cell death, DUB, proteasome, UPS

## Abstract

Programmed cell death is essential for maintaining cellular homeostasis, and its evasion is a defining hallmark of cancer. The threshold for apoptosis is largely determined by the stability of pro- and anti-apoptotic proteins, which is tightly regulated by the ubiquitin–proteasome system (UPS). In this system, E1, E2, and E3 enzymes sequentially attach ubiquitin chains that mark substrates for degradation by the 26S proteasome, whereas deubiquitinating enzymes (DUBs) reverse these modifications to stabilize specific substrates. The opposing actions of E3 ubiquitin ligases and DUBs therefore form a dynamic network that fine-tunes the abundance of key apoptotic regulators and ultimately determines cell survival or death. In this review, we summarize how DUBs control the stability of central components of both the intrinsic and extrinsic apoptotic pathways, including the p53 pathway, BCL-2 family proteins such as MCL-1, and anti-apoptotic regulators including cellular FLICE-like inhibitory protein (c-FLIP) and inhibitor of apoptosis proteins (IAPs). We also discuss how linkage-specific ubiquitin editing at death receptor complexes influences the balance between survival and apoptosis. Finally, we discuss how dysregulation of DUB-mediated protein stability promotes apoptotic evasion in cancer and highlight the therapeutic potential of DUB-targeted strategies, including catalytic inhibitors and stabilization approaches such as deubiquitinase-targeting chimeras (DUBTACs).

## 1. Introduction

Cell death is essential for maintaining cell homeostasis and preventing tumor formation. Evasion of cell death pathways allows damaged or genomically unstable cells to survive, thereby contributing to tumorigenesis [1,2]. Protein homeostasis within these pathways is predominantly regulated by the ubiquitin–proteasome system (UPS) [3], and the covalent binding of ubiquitin serves as a signal that determines the stability and functional activity of the major proteins. Deubiquitinating enzymes (DUBs) counteract this process by specifically removing ubiquitin from the target substrate [4] and act as molecular switches that regulate the balance between pro-apoptotic and anti-apoptotic factors. Dysregulation of DUBs, which play important roles in coordinating cell fate, is associated not only with malignancy but also with other diseases in which ubiquitin homeostasis is disrupted, including neurodegenerative disorders such as Parkinson’s and Alzheimer’s diseases, in which UCHL1 has been implicated, as well as inflammatory conditions [5]. In cancer, aberrant DUB activity critically influences tumor cell survival and disease progression [6]. The roles of DUBs in cancer have been extensively reviewed across a broad landscape, including comprehensive analyses of their substrate networks, catalytic mechanisms, and therapeutic potential [7,8].

Whereas these reviews survey DUB functions broadly across multiple cancer-related processes, the present review adopts an apoptotic threshold-centered framework. Rather than cataloguing DUBs individually, we examine how defined DUB–E3 regulatory relationships control the stability and activity of key apoptotic substrates and, consequently, determine the balance between cell survival and death. We integrate both the intrinsic apoptotic pathway—including the p53 axis, BCL-2 family proteins such as MCL-1, and the apoptosome—and the extrinsic pathway, in which linkage-specific ubiquitin editing at death receptor signaling complexes influences the transition between survival and apoptosis. We also highlight the regulation of anti-apoptotic proteins, particularly cellular FLICE-like inhibitory protein (c-FLIP) and inhibitor of apoptosis proteins (IAPs), which have received comparatively less attention from the DUB perspective. In addition, we evaluate the strength of the experimental evidence supporting individual DUB–substrate relationships, distinguishing direct biochemical and genetic validation from indirect, pharmacological, or correlative observations. Finally, we evaluate the therapeutic implications of these regulatory mechanisms, including the selectivity and context dependence of DUB inhibitors and the emerging potential and translational challenges of targeted protein-stabilization approaches such as deubiquitinase-targeting chimeras (DUBTACs).

The DUBs, substrates, and inhibitors discussed in this review were identified primarily through searches of PubMed and Web of Science, using combinations of terms such as “deubiquitinating enzyme” or “DUB” with “apoptosis”, “cell death”, “ubiquitin ligase”, “DUB inhibitor”, and individual DUB or substrate names, covering peer-reviewed studies published through March 2026. Priority was given to DUB–substrate relationships supported by direct biochemical or genetic evidence rather than by pharmacological or correlative data alone. This evidence-based approach enables a mechanistically focused synthesis of DUB-mediated regulation of the apoptotic threshold.

## 2. Overview of the UPS

**Figure 1:**
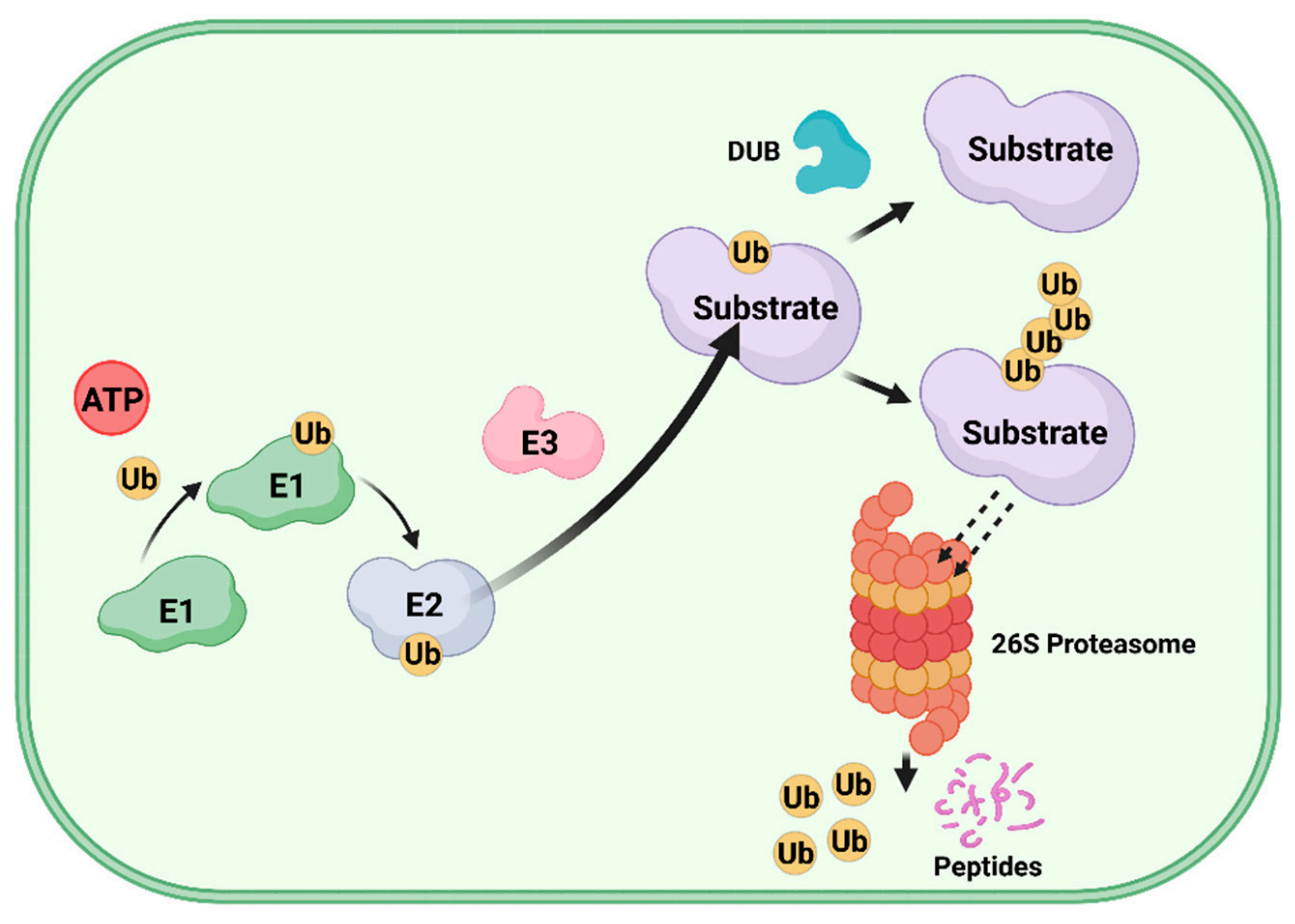
The ubiquitin–proteasome system (UPS). The ubiquitin–proteasome system is a major pathway for intracellular protein degradation. This process is initiated by ATP-dependent activation of ubiquitin (Ub) by the E1 ubiquitin-activating enzyme. The activated ubiquitin is then transferred to an E2 ubiquitin-conjugating enzyme. Next, an E3 ubiquitin ligase attaches ubiquitin to a specific target protein. Through repeated cycles of ubiquitination, multiple ubiquitin molecules form a polyubiquitin chain on the substrate protein. This chain acts as a signal for degradation. DUBs regulate this process by removing ubiquitin chains from proteins. This can stabilize the proteins and also allows ubiquitin molecules to be recycled. Finally, proteins tagged with polyubiquitin chains are recognized by the 26S proteasome and degraded into small peptides, while ubiquitin molecules are released and reused in the cell. Created in BioRender. Choi, M. (2026) https://BioRender.com/8fgrubz.

The UPS consists of three major processes: ubiquitination, deubiquitination and proteasomal degradation. In this system, a small protein called ubiquitin is covalently attached to a substrate protein destined for degradation. The resulting ubiquitin tag is recognized by the proteasome, which subsequently breaks down the target protein. Ubiquitination is mediated by a cascade of three enzymes: E1 (ubiquitin-activating enzyme), which activates ubiquitin in an ATP-dependent manner; E2 (ubiquitin-conjugating enzyme), which delivers the activated ubiquitin to the next stage; and E3 (ubiquitin ligase), which catalyzes the attachment of ubiquitin to the target protein, conferring substrate specificity (Figure 1). When multiple ubiquitin molecules are sequentially attached, a polyubiquitin chain is formed, which is recognized by the 26S proteasome, a large protease complex. Polyubiquitin chains attached to proteins exhibit linkage-specific features, and the fate of a substrate protein is largely determined by the type of linkage formed through one of the seven lysine residues or the M1 at the N-terminus. Among these, K48-linked polyubiquitin chains represent the canonical degradation signal, directing target proteins to the 26S proteasome for proteolysis. In contrast, K63-linked ubiquitin chains are primarily involved in non-proteolytic functions, such as signal transduction, endocytosis, lysosomal trafficking, and the DNA damage response. Unlike lysine-linked chains, M1-linked ubiquitin chains are formed through the N-terminal methionine and play distinct roles in signaling pathways. DUBs counteract proteolysis by removing the linkage-specific ubiquitin chains from substrate proteins before they are degraded by the proteasome. This reversible process allows DUBs to fine-tune protein stability and prolong protein lifespan in response to cellular signals. In addition to complete deubiquitination, DUBs can edit ubiquitin chains by shortening chain length or altering linkage types, thereby modulating downstream signaling outcomes. DUBs comprise nine structurally distinct subfamilies—eight cysteine-protease families and the zinc-dependent metalloprotease JAMM family—summarized in Table 1. The functional diversity of DUBs is largely defined by their specificity for different ubiquitin linkage types, which determines the fate of their substrates. For example, USP7 and OTUD5 cleave K48-linked polyubiquitin chains, the canonical signal for proteasomal degradation, and thereby regulate the stability of the tumor suppressor p53; USP7 additionally deubiquitinates its E3 ligase MDM2, so its net effect on p53 is context-dependent [9,10]. On the other hand, DUBs such as CYLD and A20 preferentially cleave K63-linked polyubiquitin chains involved in non-proteolytic signaling complexes, including the death-inducing signaling complex (DISC) and NF-κB signaling pathway, thereby modulating apoptotic thresholds and cell survival signaling. In addition, OTULIN specifically hydrolyzes M1-linked (linear) ubiquitin chains, which play a critical role in regulating inflammatory signaling and preventing aberrant cell death [11]. Collectively, these linkage-specific activities enable DUBs to function as precise molecular switches within apoptotic signaling networks.

## 3. The Canonical Apoptotic Pathways

**Figure 2:**
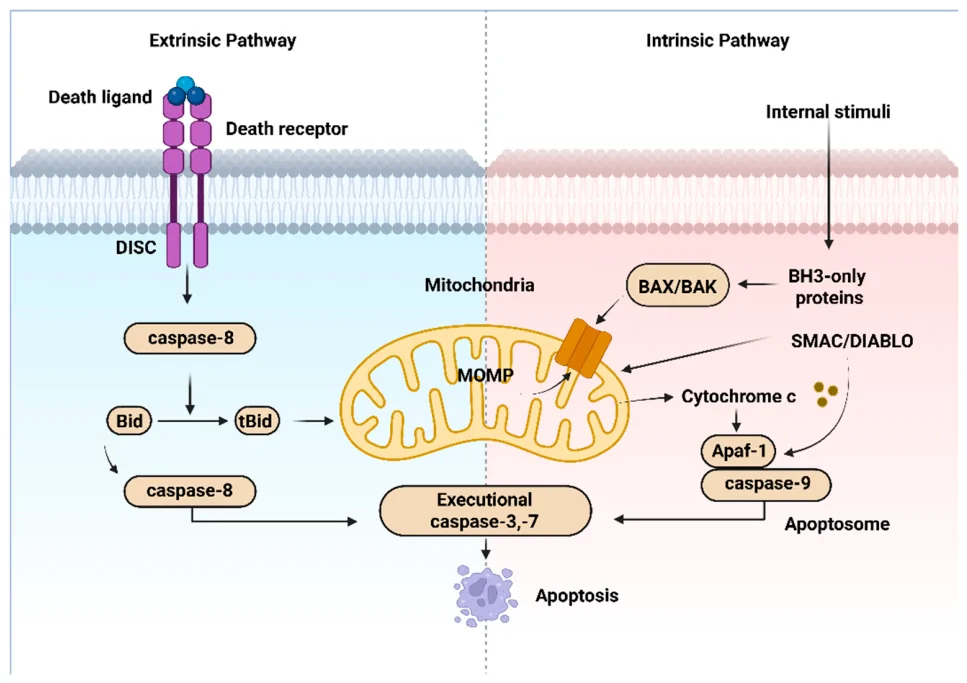
Extrinsic and intrinsic apoptotic signaling pathways. The extrinsic pathway begins when death ligands bind death receptors on the cell surface, triggering formation of the DISC and activating initiator caspase-8. Activated caspase-8 directly cleaves and activates executioner caspase-3 and caspase-7, promoting apoptosis. Caspase-8 also cleaves Bid to produce truncated Bid (tBid), which translocates to mitochondria and links the extrinsic and intrinsic pathways. The intrinsic pathway is activated by intracellular stress signals, which activate BH3-only proteins that trigger BAX- and BAK-mediated MOMP, releasing pro-apoptotic factors including cytochrome c and SMAC/DIABLO into the cytoplasm. Cytochrome c binds Apaf-1 and pro-caspase-9 to form the apoptosome complex, which activates caspase-9 and subsequently the executioner caspases, leading to programmed cell death. Created in BioRender. Choi, M. (2026) https://BioRender.com/qrxa9xa.

The canonical apoptotic pathway can be divided into intrinsic and extrinsic pathways. The intrinsic pathway is triggered by intracellular stress, such as DNA damage or oxidative stress. Pro-apoptotic proteins, including BCL-2-associated X protein (BAX) and BCL-2 homologous antagonist/killer (BAK), become activated, leading to the release of cytochrome c from the mitochondria into the cytoplasm [12]. Cytochrome c then binds to apoptotic protease-activating factor-1 (Apaf-1), forming the apoptosome. This complex activates caspases, a family of cysteine–aspartate proteases that serve as the central executioners of apoptosis, thereby initiating the apoptotic cascade. In the extrinsic pathway, binding of a death ligand to its cognate death receptor triggers the assembly of the DISC, a signaling platform that transmits extracellular death signals and directly activates caspase-8 to initiate apoptosis [13]. As shown in Figure 2, the intrinsic and extrinsic pathways converge to integrate diverse stress signals into a unified and irreversible apoptotic cascade. In the canonical apoptotic pathway, cell death proceeds in a controlled manner with a minimal inflammatory response. The extrinsic apoptotic pathway is amplified through crosstalk with the intrinsic pathway, in which caspase-8 activated in type II cells cuts Bid into truncated Bid (tBid). The resulting tBid translocates to the mitochondria and induces mitochondrial outer membrane permeabilization (MOMP) [14], thereby connecting the extrinsic and the intrinsic pathways. The oligomerized BAX and BAK form pores in the outer mitochondrial membrane, resulting in the release of apoptosis-inducing factors, including cytochrome c, into the cytoplasm. In the cytoplasm, the released cytochrome c binds to Apaf-1 in the presence of dATP, leading to the formation of a large protein complex known as apoptosome [15]. This results in the assembly of a heptameric, wheel-like protein complex that serves as a platform for pro-caspase-9 recruitment and activation. In parallel, mitochondrial permeabilization also leads to the release of SMAC/DIABLO [16], which promotes apoptosis by antagonizing IAPs, such as X-linked inhibitor of apoptosis protein (XIAP) [17], thereby further promoting progression of the caspase cascade. When pro-caspase-9 binds to the apoptosome through a CARD-CARD (caspase recruitment domain) interaction, it undergoes a structural rearrangement that results in its activation. Initiator caspases, including caspase-8 in the extrinsic pathway and caspase-9 in the intrinsic pathway, activate the executioner caspases-3, -6, and -7, which subsequently mediate the systematic dismantling of the cell [18,19].

While the canonical intrinsic and extrinsic pathways provide the fundamental framework for apoptosis, the precise threshold and execution of cell death are tightly regulated by post-translational modifications. Among these, ubiquitination and deubiquitination play critical roles in controlling the stability and activity of key apoptotic proteins. The following section discusses how the dynamic interplay between E3 ubiquitin ligases and DUBs intricately fine-tunes apoptotic signaling and ultimately determines cell fate.

## 4. DUB-Mediated Regulation of Apoptotic Protein Stability

### 4.1. DUBs Stabilizing Pro-Apoptotic and Tumor-Suppressive Proteins: The p53 Axis and BAX

At the apoptotic threshold, the tumor suppressor p53 acts as the master transcriptional activator of pro-apoptotic genes, and its abundance is determined by the opposing activities of E3 ubiquitin ligases and DUBs. USP7 directly deubiquitinates and stabilizes both the tumor suppressor p53 and its E3 ligase MDM2; because MDM2 is a major USP7 substrate under basal conditions, the net effect of USP7 activity on p53 is context-dependent. Under conditions in which MDM2 stabilization predominates, USP7 can promote p53 turnover, whereas cellular stress can shift this balance toward p53 stabilization and activation of p53-dependent transcriptional programs.

**Figure 3:**
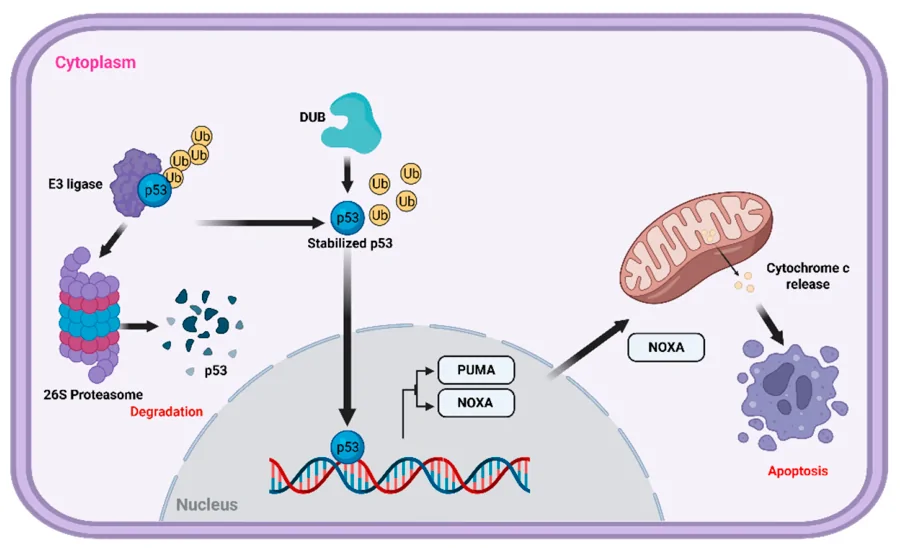
Antagonistic regulation of p53 stability by E3 ligases and DUBs and its role in apoptosis. The stability and activity of the tumor suppressor p53 are controlled by two opposing systems: E3 ubiquitin ligases and DUBs. Under normal conditions or during oncogenic stress, E3 ligases attach ubiquitin chains to p53. This poly-ubiquitination targets p53 for proteasomal degradation by the proteasome, which maintains the level of p53 low in the cell. In contrast, specific DUBs remove these ubiquitin chains from p53. This prevents its degradation and allows p53 to accumulate. Stabilized p53 then translocates to the nucleus, where it acts as a transcription factor. It increases the expression of pro-apoptotic genes such as PUMA and NOXA. These proteins promote MOMP, leading to the release of cytochrome c and activation of the apoptotic cascade. Created in BioRender. Choi, M. (2026) https://BioRender.com/at8tl43.

The OTU-family deubiquitination enzyme OTUD5 also stabilizes p53 through a direct mechanism, binding p53 and removing K48-linked ubiquitin chains, as shown by in vitro binding and deubiquitination assays, with genetic knockdown providing additional support for its role in p53 regulation [20]. OTUD5 also stabilizes PDCD5, a coactivator of p53, as demonstrated in overexpression and knockdown studies [21], suggesting an additional mechanism through which OTUD5 may enhance p53 activity. Together, these activities establish a feed-forward regulatory mechanism that amplifies p53-dependent transcriptional programs and promotes the expression of apoptotic mediators such as p21 and BAX (Figure 3). In a distinct, indirect mechanism, genetic suppression of USP5 leads to the accumulation of unanchored polyubiquitin chains, which in turn promotes p53 stabilization and enhances the transcription of its downstream target genes, including p21 and BAX [22]. Thus, DUBs can regulate p53-dependent apoptosis through both direct deubiquitination of p53 and indirect modulation of its ubiquitin-dependent regulatory environment.

The functional consequence of USP7 inhibition is more complex because USP7 regulates both p53 and its E3 ubiquitin ligase MDM2 [10,23]. Accordingly, the net effect of USP7 inhibition depends on tumor-specific variables, including the relative abundance and engagement of MDM2 and p53. In settings where MDM2 is the predominant USP7 substrate, USP7 inhibition can destabilize MDM2 and thereby enhance p53 activity, whereas substantial USP7 engagement with p53 may attenuate this effect [24]. Consistent with this mechanism, selective USP7 inhibitors such as FT671 can reactivate p53 and suppress tumor growth in vivo in mouse xenograft models [24]. TP53 mutational status is an important determinant of this response: p53 reactivation is most relevant in TP53-wild-type tumors, whereas in TP53-mutant or TP53-null tumors, USP7 inhibition may exert effects through p53-independent substrates, including N-Myc, PTEN, and DNMT1 [25,26,27]. Similarly, MDM2 amplification or overexpression may increase dependence on the USP7–MDM2 axis and thereby enhance the potential for p53 reactivation following USP7 inhibition [28]. Cellular stress can further alter this balance, as genotoxic stress has been reported to disrupt the USP7–MDM2 interaction and favor p53 stabilization [23]. In addition, the predominantly nuclear localization of the USP7–MDM2–p53 regulatory module may influence the accessibility and relative engagement of these substrates within specific cellular compartments [26]. Consequently, the therapeutic rationale for USP7 inhibition is tumor-type- and genotype-dependent rather than uniformly p53-reactivating, and biomarker-guided stratification based on TP53 status, MDM2 status, and the predominant USP7 substrate may be required to identify tumors most likely to respond through p53 restoration.

BAX, a central pro-apoptotic effector of the intrinsic pathway, represents another ubiquitin-regulated node of the apoptotic threshold. In contrast to p53, direct DUB-mediated regulation of BAX is comparatively less well characterized. BAX abundance is governed largely through indirect mechanisms, including p53-dependent transcription and through E3 ligase-mediated degradation and proteasomal degradation, notably by Parkin [29,30].

Nonetheless, a few DUBs directly regulate BAX through non-degradative ubiquitin editing rather than by controlling its stability. USP12 removes K63-linked chains from BAX to modulate its function without affecting its proteasomal turnover [31]. USP49 similarly edits non-degradative BAX polyubiquitination while additionally promoting BAX transcription [32]. The DUBs governing BAX, and the ubiquitin linkages and functional consequences involved, thus remain incompletely defined.

### 4.2. DUBs Stabilizing an Anti-Apoptotic Protein: MCL-1

Within the intrinsic apoptotic pathway, anti-apoptotic BCL-2 family proteins such as BCL-2, BCL-XL, MCL-1, and BCL-w are localized to the outer mitochondrial membrane (OMM), where they regulate MOMP [33]. These proteins promote cell survival by directly sequestering the pro-apoptotic effector proteins BAX and BAK, thereby preventing their oligomerization and pore formation [34]. They also function as molecular sinks for BH3-only activators like BIM and tBid, further suppressing apoptotic signaling. Among these anti-apoptotic proteins, MCL-1 is uniquely characterized by its exceptionally rapid turnover, making it one of the most dynamically regulated components of the apoptotic machinery [35]. Its stability is tightly controlled by the UPS, allowing MCL-1 to function as a molecular switch that rapidly adjusts cellular sensitivity to apoptotic stimuli. Under basal conditions, E3 ubiquitin ligases such as Mule/HUWE1 and FBW7 promote MCL-1 polyubiquitination and proteasomal degradation, thereby preventing its excessive accumulation and maintaining apoptotic competence [36]. The pathological elevation of Mcl-1 levels in various cancers is often attributed to the overexpression of its stabilizing DUBs or the functional impairment of the E3 ligases responsible for its degradation [37]. Impairment of E3 ligases such as Mule/HUWE1 or FBW7, which are responsible for K48-linked ubiquitination and subsequent degradation of Mcl-1 [38], often leads to the pathological accumulation of this anti-apoptotic protein, thereby conferring a significant survival advantage to malignant cells. In contrast, DUBs counteract this process to enhance cell survival. Notably, USP9X directly binds to and deubiquitinates MCL-1 in assays using endogenous proteins, while its knockdown reduces MCL-1 levels. Moreover, elevated USP9X expression correlates with MCL-1 levels in patient samples, linking the USP9X–MCL-1 axis to cancer progression [39].

Beyond USP9X, several additional DUBs have been reported to stabilize MCL-1, with varying levels of experimental support, underscoring MCL-1 as a convergence point for multiple DUBs. USP13 interacts with and deubiquitinates MCL-1 to prolong its half-life, a relationship supported by relatively strong, multi-tier evidence, including an siRNA screen, interaction and deubiquitination assays, patient-tissue correlations, and CRISPR-mediated depletion that suppresses tumor growth in lung, ovarian, and cervical cancer xenograft models [40,41]. In contrast, DUB3/USP17 and OTUD1 have been linked to MCL-1 stabilization primarily through overexpression-based or otherwise more limited evidence: DUB3/USP17 reportedly deubiquitinates MCL-1 at Lys40 and promotes chemoresistance in ovarian cancer [42], whereas OTUD1 has been reported to antagonize BH3-mimetic-induced apoptosis in cervical cancer [43]. Across these enzymes, the reported mechanisms converge on MCL-1 stabilization and increased resistance to apoptotic stimuli, although the extent to which each DUB directly counteracts specific opposing E3 ligases, including Mule/HUWE1, FBW7, and β-TrCP, remains incompletely defined. Overexpression of MCL-1 or its stabilizing DUBs leads to persistent suppression of MOMP and inhibition of cytochrome c release, even in the presence of strong apoptotic stimuli. As a result, cancer cells acquire resistance to conventional chemotherapies and targeted agents, including BH3-mimetics such as venetoclax [44]. These findings highlight the MCL-1-UPS regulatory network as a critical determinant of apoptotic sensitivity and an attractive therapeutic target in refractory malignancies.

### 4.3. DUBs Regulating Apoptosis Inhibitors: c-FLIP and IAPs

In the extrinsic apoptotic pathway, suppression of programmed cell death is largely controlled by the UPS through the regulation of c-FLIP and members of the IAP family [45]. c-FLIP functions as a critical negative regulator of death receptor signaling by interfering with the activation of caspase-8 at the DISC [46]. Specifically, c-FLIP competes with pro-caspase-8 for binding to FADD, thereby preventing caspase-8 processing and activation [47]. The cellular abundance of c-FLIP is tightly controlled by the UPS; E3 ubiquitin ligase, such as ITCH, promotes c-FLIP ubiquitination and proteasomal degradation, maintaining cellular sensitivity to death receptor-mediated apoptosis. Conversely, USP8 directly deubiquitinates and stabilizes the long isoform of c-FLIP (FLIP_L). USP8 depletion destabilizes FLIP_L, thereby promoting the assembly of DISC and TNFR1 complex II, enhancing caspase-8 and caspase-3 activation, and sensitizing cells to death receptor-induced apoptosis. USP8 is overexpressed in melanoma, cervical, and ovarian cancers, where its expression correlates with FLIP_L abundance and therapeutic resistance [48]. This USP8–FLIP_L relationship is supported by direct deubiquitination assays and genetic depletion studies, together with correlative expression data from these cancer types. The IAP family provides an additional layer of protection against apoptosis. The human IAP family comprises eight members, including XIAP, cIAP1, cIAP2, and survivin, which are characterized by one or more baculoviral IAP repeat (BIR) domains. Among these, XIAP is the only IAP that directly and potently inhibits caspases: its BIR domains suppress the execution phase of apoptosis, with BIR2 and BIR3 selectively targeting caspase-3/-7 and caspase-9, respectively [49]. XIAP stability is likewise governed by a DUB–E3 balance: USP9X removes K48-linked polyubiquitin chains from XIAP to stabilize it and confer resistance to mitotic cell death. This regulatory relationship has been validated in aggressive B-cell lymphoma using endogenous protein analyses, genetic depletion, and correlations in patient samples [50]. In contrast, cIAP1 and cIAP2 primarily function as signaling regulators rather than direct caspase inhibitors. Both proteins contain a really interesting new gene (RING) domain that confers E3 ubiquitin ligase activity [51]. Through this activity, cIAP1 and cIAP2 ubiquitinate signaling components such as RIPK1 within the TNF receptor complex, promoting pro-survival NF-κB signaling pathways while suppressing apoptotic signaling [52]. Importantly, the stability of cIAP1 and cIAP2 is autoregulated by the UPS [53]. Upon apoptotic stimulation, mitochondrial proteins such as SMAC/DIABLO are released into the cytosol and bind to IAPs. This interaction promotes autoubiquitination and proteasomal degradation of cIAP1 and cIAP2, thereby disrupting pro-survival signaling and removing an important barrier to apoptosis [54]. The stability of cIAP1 is controlled by multiple DUBs with distinct ubiquitin-linkage specificities. OTUB1 and USP19 remove K48-linked polyubiquitin chains to stabilize cIAP1, whereas USP36 preferentially removes K11-linked degradative chains from cIAP1 and K48-linked chains from survivin. Through these activities, USP36 has been reported to suppress both intrinsic and extrinsic apoptosis in colorectal cancer, in part by disrupting the XIAP–SMAC complex and promoting RIPK1 ubiquitination [55,56,57]. Notably, these DUBs appear to regulate cIAP1 through distinct mechanisms: OTUB1 and USP19 primarily affect cIAP1 abundance by removing degradative ubiquitin chains, whereas USP36 has also been implicated in modulating ubiquitin-dependent signaling, including RIPK1 ubiquitination, thereby potentially influencing signaling complex assembly in addition to protein stability. These regulatory relationships are supported primarily by biochemical deubiquitination assays and genetic manipulation, although in vivo validation across diverse tumor models remains limited. Consequently, a coordinated UPS-mediated checkpoint determines whether death receptor signaling results in cell survival or apoptotic cell death. Elevated levels of c-FLIP and IAPs, sustained by DUB-mediated stabilization, are associated with resistance to death-receptor-induced apoptosis and poor clinical outcomes in several cancers.

The endogenous IAP-antagonist function of SMAC/DIABLO has been therapeutically exploited through the development of Smac-mimetics, small molecules that mimic the N-terminal AVPI tetrapeptide motif of SMAC and bind to the BIR domains of IAPs. By occupying these domains, Smac-mimetics antagonize XIAP-mediated inhibition of caspase-3, -7, and -9 and induce the autoubiquitination and subsequent proteasomal degradation of cIAP1 and cIAP2. Loss of cIAP1/2 promotes the formation of a RIPK1–caspase-8 signaling complex and activation of non-canonical NF-κB signaling, thereby sensitizing tumor cells to TNF-driven apoptosis [58]. Several Smac-mimetics, including birinapant and LCL161, have advanced to clinical evaluation; however, their limited single-agent efficacy has highlighted the importance of combination strategies and appropriate patient selection [59]. Notably, because the stability of cIAP1/2 is also governed by DUBs such as OTUB1, USP19, and USP36, DUB activity may influence the extent of cIAP1/2 degradation induced by Smac-mimetics and, consequently, cellular sensitivity to these agents.

### 4.4. Linkage-Specific Ubiquitin Editing at the TNFR Complex

In the extrinsic apoptotic pathway, dynamic ubiquitination within the tumor necrosis factor receptor (TNFR) signaling complex plays a critical role in determining cell fate [60]. Upon TNF-α stimulation, TNFR1 recruits adaptor proteins, TRADD, RIPK1, TRAF2, and cIAP1/2, to assemble Complex I at the plasma membrane [61]. Within this complex, cIAP1 and cIAP2 catalyze K63-linked polyubiquitination of RIPK1. This modified RIPK1 recruits the linear ubiquitin chain assembly complex (LUBAC), consisting of HOIP, HOIL-1L, and SHARPIN. LUBAC subsequently catalyzes M1-linked (linear) ubiquitination of RIPK1 and NF-κB essential modulator (NEMO). These linear ubiquitin chains promote IκB kinase (IKK) complex activation and canonical NF-κB signaling, resulting in the transcriptional upregulation of pro-survival genes, including c-FLIP and cIAPs, and thereby favoring cell survival [62]. Conversely, disruption of this signaling network shifts the cellular response toward apoptosis. When LUBAC activity is impaired [11] or the K63- and M1-linked chains on RIPK1 are removed by CYLD, Complex I becomes unstable. These DUBs are not functionally equivalent: CYLD hydrolyzes both K63- and M1-linked ubiquitin chains, whereas OTULIN is highly specific for M1-linked (linear) chains. A20 is a multifunctional OTU family enzyme that removes K63-linked ubiquitin chains and also binds M1-linked chains, thereby exerting distinct regulatory effects on TNFR1 signaling [63,64]. These DUBs also differ in their recruitment to and mechanisms of action within the TNFR1 signaling complex. CYLD is recruited through a SPATA2-mediated interaction with the LUBAC subunit HOIP, whereas OTULIN binds directly to HOIP via its PIM motif but is not efficiently recruited to the receptor complex. A20, in contrast, is recruited and stabilized at the complex through its interaction with M1-linked ubiquitin chains. All three enzymes are cysteine-protease DUBs; however, OTULIN employs a substrate-assisted catalytic mechanism in which the M1-linked ubiquitin substrate itself contributes to formation of the productive active site, accounting for its strict linkage specificity. Consequently, these DUBs differ not only in their ubiquitin-linkage preference but also in their molecular mechanisms and downstream biological consequences. Functionally, CYLD removes these chains from RIPK1 and thereby promotes its release into Complex II, whereas A20 restrains this transition, and OTULIN acts mainly on M1-chain homeostasis rather than on RIPK1 within the receptor complex. Within Complex II, caspase-8 is activated and the extrinsic apoptotic pathway is initiated. The outcome at Complex II is further determined by RIPK1 phosphorylation, c-FLIP abundance, and caspase-8 activity. When caspase-8 is inhibited and RIPK3 and MLKL are available, RIPK1 can engage RIPK3 and promote necroptosis instead of apoptosis [65]. Thus, the dynamic balance between LUBAC-mediated linear ubiquitination and DUB-mediated deubiquitination serves as a critical molecular switch that determines whether TNF signaling promotes cell survival or apoptotic cell death [62].

## 5. Therapeutic Targeting of DUBs to Restore Apoptotic Ability

Small molecules can directly modulate protein–protein interactions involved in apoptosis, thereby controlling cancer cell survival and death [66]. Apoptotic proteins interact through specific amino acid motifs, and small molecules can competitively bind to these sites before endogenous binding partners, preventing or disrupting these interactions [67]. A representative class is BH3 mimetics, which are pharmacologically engineered to occupy the BH3-binding hydrophobic grooves of anti-apoptotic proteins (e.g., BCL-2, BCL-XL, and MCL-1) [68]. By doing so, they prevent these anti-apoptotic proteins from sequestering pro-apoptotic effectors such as BAX and BAK or displace BIM and other BH3-only activators to trigger MOMP [69]. Unlike large biologics, small molecules can access narrow, deep-seated pockets at PPI interfaces that are otherwise sterically hindered, making them more effective at disrupting these interactions. Small molecules can intervene in the UPS to control protein homeostasis. A well-known example is the inhibition of p53 degradation through blockade of MDM2. Under normal conditions, MDM2 binds to p53 and promotes its ubiquitination and proteasomal degradation [70]. Small molecules such as Nutlin competitively bind to MDM2, preventing its interaction with p53. As a result, p53 is stabilized and accumulates within the cell, leading to increased transcription of pro-apoptotic genes such as BAX, PUMA, and NOXA, and ultimately promoting apoptosis [71].

While occupancy-based inhibitors have achieved notable success, the cell’s ability to compensate through protein upregulation or mutations often limits their sustained effectiveness. This has led to the development of PROTACs, which offer a different tactical approach by leveraging the UPS to degrade, rather than simply blocking the target. Because PROTACs act catalytically to remove anti-apoptotic proteins from the cell entirely, they can overcome many of the resistance mechanisms that plague traditional small molecules. The emergence of PROTAC technology represents a paradigm shift in targeting apoptotic regulators. These heterobifunctional small molecules recruit E3 ubiquitin ligases to induce the selective proteasomal degradation of previously ‘undruggable’ anti-apoptotic proteins and, in some contexts, provide more sustained inhibition than traditional occupancy-based inhibitors that require continuous target engagement. A conceptually related IAP-based strategy is provided by SNIPERs (specific and nongenetic IAP-dependent protein erasers), chimeric small molecules that recruit the E3 ligase activity of IAPs, including cIAP1, cIAP2, and XIAP, to promote ubiquitination and proteasomal degradation of target proteins [72]. Unlike PROTACs, which typically recruit E3 ligases such as VHL or CRBN, SNIPERs exploit IAPs as the recruited E3 machinery, thereby linking targeted protein degradation to the intrinsic regulatory functions of apoptosis-associated ubiquitin ligases. Importantly, some SNIPERs can also induce degradation of the recruited IAP itself, particularly cIAP1, through autoubiquitination, potentially producing a dual effect of target protein degradation and attenuation of IAP-mediated survival signaling. Because IAPs are frequently dysregulated or overexpressed in cancer, this property may provide an additional therapeutic advantage by simultaneously reducing the abundance or function of anti-apoptotic IAPs and eliminating selected oncogenic or disease-associated proteins. However, the efficacy of SNIPERs remains dependent on the availability, cellular localization, and functional competence of the recruited IAP, as well as productive ternary-complex formation. Thus, the biological effects of SNIPERs may reflect both target degradation and modulation of IAP-dependent apoptotic signaling, highlighting the importance of carefully distinguishing these mechanisms when evaluating their therapeutic activity.

In contrast, DUBTACs stabilize target proteins by recruiting a DUB to remove degradative ubiquitin chains, thereby opposing rather than promoting degradation [73]. This strategy is conceptually attractive for restoring tumor suppressors that are destabilized in cancer; however, its current limitations warrant critical assessment beyond the basic DUBTAC concept. First, the repertoire of DUBs that have been experimentally demonstrated to be effectively recruited remains limited. Proof-of-concept DUBTACs have relied predominantly on OTUB1, which can be engaged by a covalent ligand targeting a non-catalytic cysteine [73], whereas validated ligands for other DUBs remain scarce. Second, DUBTAC activity is likely to depend strongly on the identity and ubiquitination state of the target substrate, as well as on subcellular compartmentalization. OTUB1 preferentially regulates K48-linked ubiquitin chains and is predominantly localized in the cytosol and nucleus; thus, productive target stabilization requires the target to carry accessible K48-linked ubiquitin chains and to be brought into sufficient spatial proximity to OTUB1. These requirements may limit the applicability of DUBTACs to substrates with distinct ubiquitin architectures or restricted subcellular localization. Third, the requirement for catalytic activity may vary among DUBTAC systems. In the case of OTUB1, productive target stabilization depends on enzymatic removal of ubiquitin chains, which is mechanistically distinct from its non-catalytic inhibition of E2 enzymes [74,75]. More broadly, recruitment of a DUB to a target does not by itself guarantee productive deubiquitination or protein stabilization, as the outcome depends on the specific DUB–substrate interaction, ubiquitin linkage context, catalytic competence, and spatial organization of the ternary complex. Fourth, the available evidence remains largely at proof-of-concept stage, with studies involving a limited number of target proteins and relatively limited validation in cancer models [76]. Finally, clinical translation is constrained by several challenges, including the availability of selective DUB ligands, productive ternary complex formation, appropriate recognition of target ubiquitin linkages, target and subcellular compartment dependence, cellular delivery and permeability, potential on-target effects arising from DUB recruitment to unintended substrates, and the current absence of clinical-stage DUBTACs. Nevertheless, the ability to restore the stability and function of destabilized tumor suppressors, rather than simply inhibiting oncogenic proteins, represents a distinct therapeutic opportunity in apoptosis-targeted cancer therapy if these mechanistic and translational barriers can be overcome.

DUBTACs are one of several emerging strategies designed to increase, rather than decrease, the level or activity of a target protein [76]. RESTORACs (Stablix Therapeutics) were developed with a similar goal of restoring the level and function of proteins that undergo excessive degradation in disease, whereas ENTACs (enhancement-targeting chimeras; Entact Bio) extend this concept beyond protein abundance by aiming to restore or enhance protein function, including through correction of aberrant subcellular localization or activity. These modalities are not mechanistically equivalent, as they differ in their intended outcomes, substrate requirements, and chemical architectures. The level of supporting evidence also differs among these approaches. DUBTACs have been demonstrated in proof-of-concept studies involving a limited number of substrates, whereas RESTORACs and ENTACs remain emerging platform technologies with preclinical evidence, and none has yet entered clinical trials. By restoring proteins that are lost or functionally impaired, these approaches are conceptually complementary to degradation-based modalities such as PROTACs and SNIPERs. This distinction is particularly relevant to regulation of the apoptotic threshold, because apoptotic evasion can result both from the accumulation or stabilization of anti-apoptotic proteins, such as MCL-1, c-FLIP, and IAPs, and from excessive degradation or functional loss of pro-apoptotic or tumor-suppressive proteins, such as p53 and BAX.

Pharmacological targeting of DUBs has emerged as a distinct modality for inducing apoptosis by directly manipulating the cellular degradation machinery [77]. Unlike traditional occupancy-based antagonists, DUB inhibitors lower the absolute abundance of target proteins, thereby bypassing the feedback loops and competitive binding constraints typical of standard inhibitors. The USP7–MDM2–p53 axis illustrates this mechanism: in TP53–wild-type contexts, USP7 inhibition promotes MDM2 autoubiquitination and degradation, thereby stabilizing p53 and driving the transcriptional activation of pro-apoptotic effectors such as BAX and PUMA [78]. However, because USP7 also directly deubiquitinates p53 and regulates numerous p53-independent substrates, these effects are context-dependent rather than universal, as discussed above. This regulatory scope extends to direct apoptotic threshold modulators like USP9X, which deubiquitinates and stabilizes MCL-1 and XIAP [50]. Pharmacological inhibition with the broad-spectrum DUB inhibitor WP1130 has been reported to reduce MCL-1 levels and sensitize refractory cancer cells to BCL-2 antagonists [79]. However, WP1130 is not selective for USP9X and has been reported to inhibit multiple DUBs, including USP9X, USP5, USP14, and UCHL5 [77]. Therefore, changes in MCL-1 abundance or apoptotic sensitivity following WP1130 treatment cannot be attributed specifically to USP9X without additional genetic or biochemical validation. Among these targets, the proteasome-associated DUBs USP14 and UCHL5 can also be inhibited by agents such as VLX1570 or b-AP15, which interfere with ubiquitin recycling at the 19S regulatory particle. Disruption of proteasomal ubiquitin processing can impair protein flux and induce proteotoxic stress, a vulnerability that is particularly pronounced in highly secretory malignancies such as multiple myeloma [80]. Thus, the apoptotic sensitization observed following WP1130 treatment may reflect the combined inhibition of multiple DUBs, including proteasome-associated DUBs, rather than USP9X blockade alone. However, clinical translation has proven challenging. VLX1570, one of the most advanced proteasome-associated DUB inhibitors, was discontinued after two patients treated at the 1.2 mg/kg dose level in a Phase 1 trial in relapsed and/or refractory multiple myeloma developed severe, abrupt, and progressive respiratory insufficiency with diffuse pulmonary infiltrates that culminated in death [81]. This experience highlights the difficulty of achieving sufficient antitumor activity while preserving an acceptable therapeutic window when targeting proteasome-associated ubiquitin processing.

**Figure 4:**
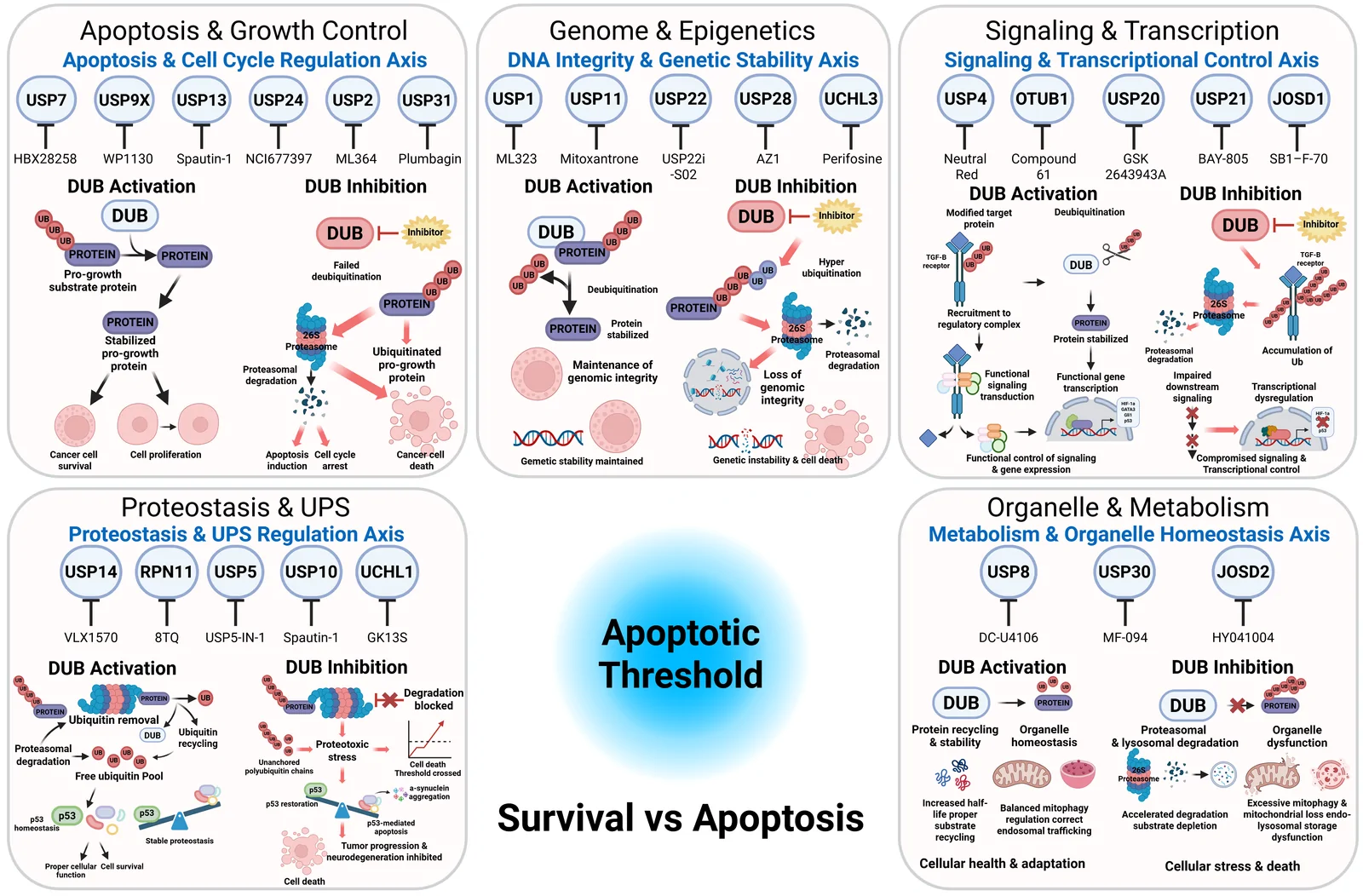
DUB-mediated control of the apoptotic threshold. Representative deubiquitinating enzymes (DUBs) and their small-molecule inhibitors are shown across five functional axes that regulate the apoptotic threshold. Each DUB influences the stability or signaling activity of its relevant substrate through deubiquitination, and the balance between DUB activity and inhibition shifts cells toward survival or apoptosis. These axes act in parallel to determine the apoptotic threshold rather than converging on a single node such as p53. Created in BioRender. Choi, M. (2026) https://BioRender.com/ywnafot.

**Figure 5:**
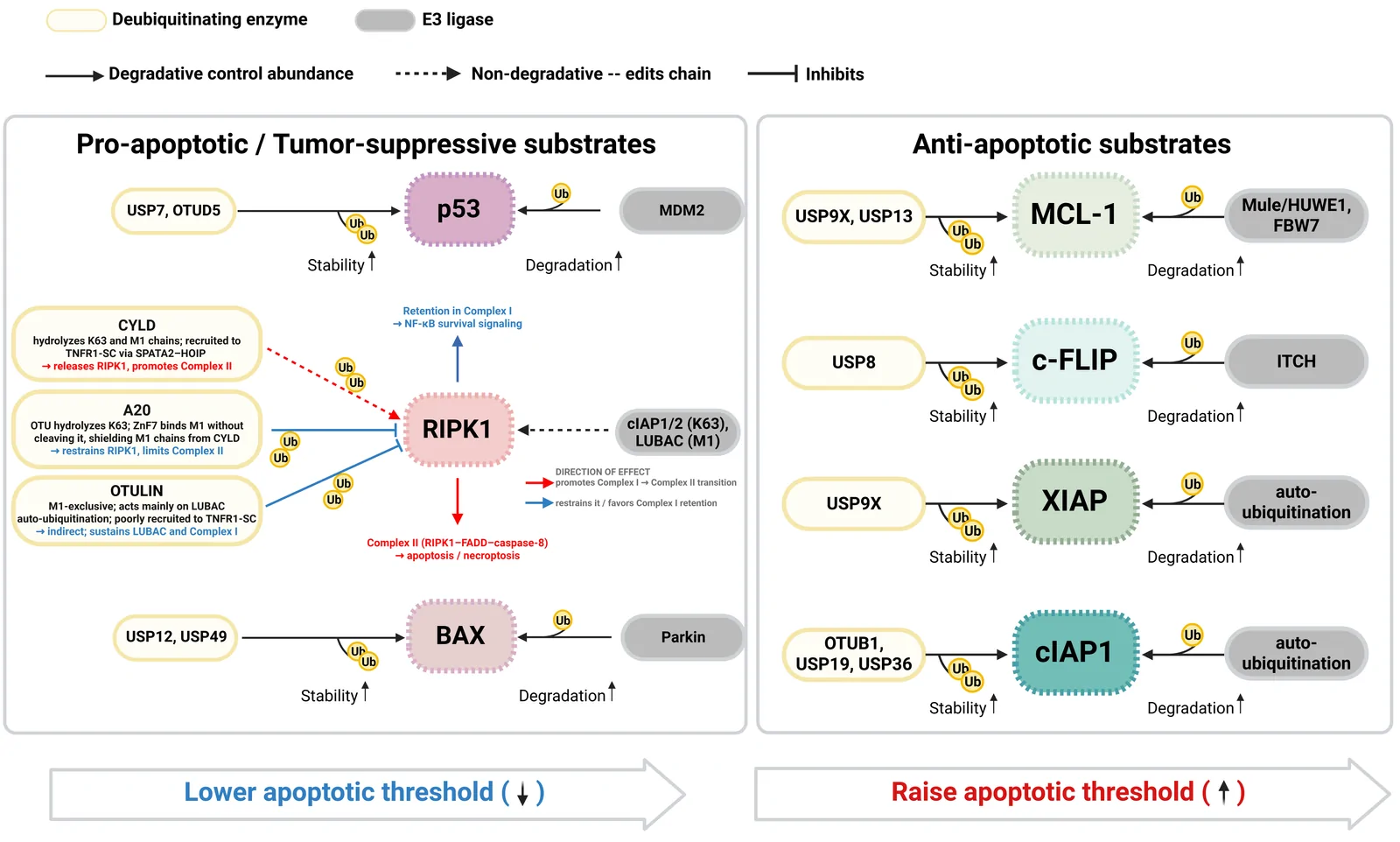
Opposing DUB and E3 ligase activities control the apoptotic threshold through substrate stability and ubiquitin-dependent signaling. Each substrate (center) is regulated by a DUB (**Left**) and, where established, an opposing E3 ligase or autoubiquitination mechanism (**Right**). The balance between these activities influences substrate abundance or signaling activity and thereby shifts the apoptotic threshold. Solid arrows denote degradative regulation that controls substrate abundance, dashed arrows denote non-degradative ubiquitin editing that modulates protein function or localization, and blunt-ended lines denote restraint of RIPK1 activation and of the transition to Complex II. In the RIPK1 axis, cIAP1/2- and LUBAC-generated K63- and M1-linked chains retain RIPK1 within TNFR1 Complex I and support NF-κB-dependent survival signaling. Because RIPK1 is regulated predominantly through non-degradative ubiquitin signaling rather than degradative turnover, this axis is depicted in terms of the Complex I–Complex II transition rather than changes in protein stability. CYLD, A20, and OTULIN are shown separately to reflect their distinct mechanisms. CYLD, which is recruited to the receptor complex via SPATA2–HOIP, removes K63- and M1-linked chains from RIPK1 and promotes its release into cytosolic Complex II, thereby favoring apoptotic or necroptotic signaling. A20 restrains this transition through K63-chain hydrolysis together with non-catalytic, ZnF7-mediated binding to M1-chains. OTULIN is an M1-specific DUB that associates with HOIP through its PUB-interacting motif and acts primarily in M1-chain homeostasis; it is not efficiently recruited to the TNFR1 receptor complex and therefore does not directly regulate RIPK1 within the receptor-associated complex. Created in BioRender. Choi, M. (2026) https://BioRender.com/gmkz9jq.

Beyond the apoptosis-centered mechanisms discussed above, DUBs also regulate the stability of proteins involved in genome maintenance, transcriptional regulation, and organelle quality control. Pharmacological inhibition of these DUBs, including USP1, USP28, USP8, and USP30, can destabilize key pro-survival substrates and thereby indirectly lower the apoptotic threshold. USP1 illustrates that the therapeutic relevance of DUB inhibition is determined by specific molecular contexts rather than by a general capacity to promote cell death. Through deubiquitination of monoubiquitinated PCNA and FANCD2, USP1 regulates translesion synthesis and the Fanconi anemia pathway, thereby supporting tolerance to replication stress and homologous recombination (HR)-mediated DNA repair [82,83]. Accordingly, USP1 inhibition may be particularly relevant in tumors characterized by homologous recombination deficiency (HRD) or increased replication stress and may provide a strategy to overcome or delay resistance to PARP inhibitors [84]. Thus, the therapeutic rationale for USP1 inhibition is better understood in the context of tumor-specific DNA damage response dependencies rather than as a nonspecific means of increasing cell death. As illustrated in Figure 4, these regulatory pathways act in parallel to determine the apoptotic threshold through both p53-dependent and p53-independent axes, including MCL-1, c-FLIP, XIAP, RIPK1, and proteotoxic stress, thereby collectively influencing the balance between cell survival and apoptosis. Because a comprehensive discussion of each non-apoptotic pathway is beyond the scope of this review, we focus primarily on DUBs with defined mechanistic links to the apoptotic threshold. These include DUBs that directly deubiquitinate apoptotic regulators, such as USP7 and OTUD5 in the p53 pathway and USP9X in the regulation of MCL-1 and XIAP, as well as DUBs that act indirectly, such as USP5, which promotes p53 stabilization through the accumulation of unanchored polyubiquitin chains rather than through direct deubiquitination of p53 [22], and the proteasome-associated DUBs USP14 and UCHL5, which modulate apoptotic sensitivity through global proteostasis and proteotoxic stress. The broader landscape of DUB substrates and their corresponding inhibitors is summarized in Figure 4 and Table 2. Figure 5 complements this view by mapping the individual DUB–substrate–E3 ligase axes discussed above, distinguishing DUBs that control substrate abundance from those that edit non-degradative ubiquitin chains and thereby modulate protein function or signaling, as exemplified by BAX and RIPK1.

## 6. Conclusions

In conclusion, this review highlights that the opposing activities of E3 ubiquitin ligases and DUBs constitute a decisive regulatory layer governing apoptotic protein stability, and that cancer cells frequently exploit this balance to evade programmed cell death. Notably, the biological function of a given DUB is highly context-dependent rather than uniformly pro- or anti-apoptotic, as its effects are determined by the specific substrate it regulates and the surrounding cellular environment. This functional complexity, together with the high structural conservation of DUB catalytic domains, presents a major challenge for the development of substrate-selective inhibitors. Moreover, catalytic inhibition alone may be insufficient to abolish the non-catalytic or scaffolding functions that some DUBs retain independently of their enzymatic activity. Overcoming these barriers will require comprehensive proteome-wide identification of DUB substrates, linkage- and site-specific ubiquitination analyses, and biomarker-guided patient stratification to identify tumors most likely to benefit from DUB-targeted therapies. In parallel, both catalytic inhibitors and targeted protein stabilization strategies, such as DUBTACs, pose distinct design challenges. Reflecting these unresolved challenges, relatively few DUB-targeted agents have advanced to clinical development, underscoring that this field remains at an earlier stage than other therapeutic approaches targeting the UPS. Continued efforts to resolve DUB substrate specificity and context-dependent function will be essential for fully realizing the potential of DUBs as precision therapeutic targets in cancer.

## Figures and Tables

**Table 1:** Structural and functional taxonomy of the human deubiquitinating enzyme (DUB) superfamily.

Catalytic Class	Family	Full Name	Mechanisms & Characteristics	Key Examples
Cysteine protease	USP	Ubiquitin-specific protease	The largest and most diverse DUB family; characterized by highly divergent structures and varying substrate specificities.	USP1, USP2, USP3, USP4, USP5, USP6, USP7, USP8, USP9X, USP9Y, USP10, USP11, USP12, USP13, USP14, USP15, USP16, USP17, USP18, USP19, USP20, USP21, USP22, USP24, USP25, USP26, USP27X, USP28, USP29, USP30, USP31, USP32, USP33, USP34, USP35, USP36, USP37, USP38, USP39, USP40, USP41, USP42, USP43, USP44, USP45, USP46, USP47, USP48, USP49, USP50, USP51, USP53, USP54, USPL1, CYLD
	UCH	Ubiquitin C-terminal hydrolase	Primarily involved in processing small ubiquitin adducts or precursors; generally favors small, unstructured substrates.	UCH-L1, UCH-L3, UCH-L5, BAP1
	OTU	Ovarian tumor protease	Exhibits high linkage specificity (e.g., K48, K63) towards polyubiquitin chains, precisely regulating signaling pathways.	OTUD1, OTUD2, OTUD3, OTUD4, OTUD5, OTUD6A, OTUD6B, OTUD7A, OTUD7B, OTUB1, OTUB2, OTULIN, TNFAIP3, VCPIP1, ALG13, ZRANB1
	MJD	Machado–Josephin domain	Acts through a conserved Josephin domain; often associated with the regulation of neurodegenerative disease-related proteins.	ATXN3, ATXN3L, JOSD1, JOSD2
	MINDY	Motif interacting with Ub-containing novel DUB family	Highly selective for the cleavage of K48-linked polyubiquitin chains; plays a critical role in regulating protein degradation.	MINDY1, MINDY2, MINDY3, MINDY4, MINDY4B
	ZUP1	Zinc finger-containing Ub-specific peptidase 1	A recently identified family involved in DNA damage repair and the regulation of K63-linked ubiquitin chains.	ZUP1
	PPPDE	Permuted papain fold peptidases of dsRNA viruses and eukaryotes	A novel DUB group with a papain-like fold, possessing both deubiquitinating and deISGylating activities.	PPPDE1, PPPDE2
	MCPIP	Monocyte chemotactic protein-induced protein	Also known as Regnase-1; orchestrates immune responses and cellular fate through dual DUB and RNase activities.	MCPIP1
Metalloproteases	JAMM	JAB1/MPN/MOV34 metalloenzyme	Utilizes a Zn^2+^-dependent catalytic mechanism; typically functions as a critical component of large multi-subunit complexes.	PSMD14, PSMD7, COPS5, COPS6, STAMBP, STAMBPL1, BRCC3, MYSM1, MPND, EIF3F, EIF3H, PRPF8

**Table 2:** Experimental DUB-targeting agents relevant to apoptosis regulation, comprising small-molecule inhibitors and one engineered ubiquitin variant.

Targeted DUBs	Small Molecules	Class/Selectivity	Mechanism of Action	References
USP7 (HAUSP)	HBX28258, HBX41108, GNE6640, GNE6776, I-56, I-57, DC-U43-10, P5091, P22077, FT671, FT827, XL177A	Selective: XL177A, FT671, FT827, GNE-6640, GNE-6776. Non-selective: HBX41108, P5091, P22077. Selectivity not established: HBX28258, I-56, I-57, DC-U43-10.	Restores p53 stability by promoting MDM2 auto-ubiquitination and degradation	[10,24,28,85,86,87,88,89,90,91]
USP9X	WP1130 (Degrasyn), G9	Non-selective: WP1130 (also inhibits USP5, USP14, and UCHL5) ^1^; G9/EOAI3402143 (also inhibits USP24 and USP5; the authors report that USP5 inhibition could not be eliminated) ^2^.	Induces proteasomal degradation of MCL-1 by inhibiting its deubiquitination	[39,77,92,93,94]
USP14/UCHL5	b-AP15, VLX1570, IU1, IU1-47, IU1-248	IU1 and its analogues (IU1-47, IU1-248) (reversible, USP14-directed inhibitors reported to show selectivity over other DUBs, although the scope of counter-screening differs between studies) ^3^. Non-selective: b-AP15 and VLX1570 (structurally related α,β-unsaturated carbonyl compounds that inhibit both USP14 and UCHL5 and additionally cross-link multiple unrelated proteins; VLX1570 exhibited fatal dose-limiting pulmonary toxicity in early clinical evaluation) ^4^.	Promote proteotoxic stress by blocking ubiquitin recycling via proteasome-associated DUB inhibition	[95,96,97,98,99,100,101,102]
USP1	ML323, SJB2-043, GW7647, KSQ-4279	Selective: ML323 (>1500-fold selectivity over USP2, USP5, USP7, USP8, and USP46/UAF1; no significant activity in a 451-kinase panel) ^5^; KSQ-4279 (clinical-stage, first-in-class USP1 inhibitor). Non-selective: GW7647 (repurposed PPARα agonist; also inhibits USP12) ^6^. Selectivity not established: SJB2-043.	Inhibits deubiquitination of DDR proteins, increasing susceptibility to cell death	[82,83,84,103,104,105,106]
USP10	Spautin-1, UbV.10.1, Wu-5	Selective: UbV.10.1 (engineered ubiquitin variant, not a small molecule) ^7^. Non-selective: Spautin-1 (dual USP10/USP13 inhibitor that also inhibits mitochondrial complex I; silencing of USP10 and USP13 does not reproduce its cellular effects) ^8^. Selectivity not established: Wu-5.	Regulates the apoptotic threshold via autophagy and p53 homeostasis	[107,108,109,110]
USP28	Compound 9p, Compound 19, Compound 29, Compound 58, AZ1-AZ4, FT206	Non-selective: AZ1–AZ4 and FT206 (dual USP25/USP28 inhibitors; FT206 shows a high degree of selectivity over other DUBs but cross-reacts with its closest homologue USP25) ^9^. Selectivity not established: Compound 19 (counter-screened only against USP7 and LSD1; selectivity versus USP25 not assessed), Compound 9p, Compound 29, Compound 58.	Promotes degradation of c-Myc and 53BP1, suppressing oncogenic signaling and DNA repair	[111,112,113,114,115,116,117,118]
USP2	LCAHA, LCAE, ML364, COH29, 6-TG	Selective: ML364 ^10^. Non-selective: COH29 (repurposed ribonucleotide reductase inhibitor); 6-TG (repurposed thiopurine antimetabolite); LCAHA and LCAE (bile acid derivatives showing preferential activity toward the USP family in a 32-DUB panel rather than USP2a-restricted inhibition) ^11^.	Promotes degradation of Cyclin D1, MDM2, and FASN via deubiquitination blockade	[119,120,121,122,123]
USP4	Neutral Red, U4-I05	Selectivity not established: Neutral Red (repurposed phenazine dye; reported to inhibit USP4 without activity against YOD1, USP5, UCHL1, or USP14, but only at IC_50_ ≈ 50 μM) ^12^; U4-I05 (off-target assessment limited to a single indirect readout for USP46) ^13^.	Suppresses Wnt/β-catenin signaling and cancer stemness	[124,125]
USP5	USP5-IN-1	Selectivity not established: USP5-IN-1 (a ligand of the non-catalytic USP5 zinc-finger ubiquitin-binding domain, KD ≈ 2.8 μM; no DUB panel profiling reported) ^14^.	Promotes p53 stabilization; USP5 inhibition leads to accumulation of unanchored polyubiquitin chains, which interfere with proteasomal degradation of p53.	[22,126,127]
USP8	DC-U4106, Compound 61	Non-selective: Compound 61 (dual OTUB1/USP8 inhibitor; see also the OTUB1 entry) ^15^. Selectivity not established: DC-U4106 (reported IC_50_ 1.2 μM for USP8, counter-screened against USP2 and USP7 only) ^16^.	Suppresses RTK (EGFR) signaling by inhibiting endosomal recycling/trafficking	[128,129,130]
USP11	Mitoxantrone	Non-selective: Mitoxantrone (an approved anthracenedione chemotherapeutic whose primary target is type II topoisomerase; identified as a USP11 inhibitor by screening rather than by design) ^17^.	Compromises DNA damage repair by inhibiting BRCA2/PALB2 stabilization	[131]
USP13	Spautin-1	Non-selective: Spautin-1 (dual USP10/USP13 inhibitor that also inhibits mitochondrial complex I) ^18^.	Reduces MCL-1 and PTEN stability, sensitizing cells to apoptosis	[40,107,132]
USP20	GSK2643943A	Selectivity not established: GSK2643943A (reported USP20 IC_50_ ≈ 160 nM; no primary characterization report describing its DUB selectivity was identified) ^19^.	Regulates hypoxic responses and DNA damage signaling (HIF-1α, Rad17)	[133,134]
USP21	BAY-805	Selective: BAY-805 (IC_50_ < 10 nM against USP21, counter-screened against a 10-member USP panel, with cellular target engagement at EC_50_ ≈ 95 nM and a structurally matched inactive control compound, BAY-728) ^20^.	Disrupts stability of GATA3 and Gli1, impairing immune differentiation and oncogenic signaling	[135]
USP22	Baohuoside I, Morusin, Chrysin, USP22i-S02	Non-selective: Baohuoside I, Morusin, and Chrysin (flavonoid natural products identified by docking and cellular histone ubiquitination readouts at 8–40 μM; Baohuoside I is also reported as a CXCR4 antagonist) ^21^. Selectivity not established: USP22i-S02 (no DUB panel profiling reported).	Blocks deubiquitination of histone H2B, Sirt1, Foxp3, reprogramming oncogenic progression/immunity	[136,137]
USP24	NCI677397	Selectivity not established: NCI677397 (no DUB selectivity data reported in the primary study)	Induces ferroptosis by destabilizing GPX4, elevating lipid peroxidation	[138,139]
USP31	Plumbagin	Non-selective: Plumbagin (a naphthoquinone natural product described in the primary report as an inhibitor of deubiquitinating enzymes with preference for USP31) ^22^.	Promotes ubiquitination and proteasomal degradation of GPX4, inducing apoptosis in hepatocellular carcinoma	[140]
USP30	15-oxospiramilactone, MF-094, Compound 39	Selective: Compound 39 (no significant activity against 49 other endogenous DUBs profiled by activity-based protein profiling mass spectrometry in SH-SY5Y cells) ^23^; MF-094 (USP30 IC_50_ ≈ 120 nM; <30% inhibition of 22 other DUBs at 10 μM) ^24^. Selectivity not established: 15-oxospiramilactone (S3), a covalent natural product targeting the catalytic cysteine of USP30 for which no DUB panel profiling has been reported ^25^.	Enhances mitophagy and mitochondrial clearance via ubiquitination of outer mitochondrial membrane proteins	[141,142,143,144,145]
UCHL1	LDN-57444, LDN-91946, IMP-1710, GK13S	Selective: IMP-1710 ^26^; GK13S ^27^; LDN-91946 (no inhibition of UCH-L3, isopeptidase T, papain, caspase-3, or tissue transglutaminase) ^28^. Selectivity not established: LDN-57444 ^29^.	Disrupts ubiquitin homeostasis and α-synuclein/p53 stability	[146,147,148,149]
UCHL3	Perifosine, TCID	Non-selective: Perifosine (a clinically evaluated alkylphospholipid AKT-pathway inhibitor for which UCHL3 inhibition was identified as an additional activity) ^30^. Selectivity not established: TCID (UCH-L3 IC_50_ ≈ 0.6 μM with >100-fold selectivity over UCH-L1, but no broader DUB panel profiling reported) ^31^.	Modulates DNA repair and insulin signaling via RAD51/GLUT4 stability	[147,150,151,152]
RPN11 (PSMD14)	8TQ, Capzimin, SOP11, Thiolutin	Non-selective: Capzimin (>5-fold selectivity over related JAMM proteases only, with >2-log selectivity limited to structurally unrelated metalloenzymes) ^32^; Thiolutin (a zinc chelator that also inhibits CSN5, AMSH, and BRCC36) ^33^; SOP11 (an epidithiodiketopiperazine; compounds of this class also inhibit CSN5 and AMSH) ^34^. Selectivity not established: 8TQ.	Inhibits proteasomal degradation by blocking ubiquitin removal prior to translocation	[153,154,155,156]
JOSD1	SB1-F-70	Non-selective: SB1-F-70 (DUBome profiling in the primary report showed inhibition of USP30 and UCHL1 in addition to JOSD1) ^35^.	Induces death of JAK2-V617F leukemia cells by promoting mutant JAK2 degradation	[157]
JOSD2	HY041004	Non-selective: HY041004 (inhibits JOSD1 with comparable potency; IC_50_ 0.26 μM for JOSD2 versus 0.47 μM for JOSD1) ^36^.	Suppresses NSCLC by restoring LKB1 activity (K6-linked deubiquitination)	[158,159]
OTUB1	Compound 61 (OTUB1/USP8-IN-1)	Non-selective: Compound 61 (dual OTUB1/USP8 inhibitor; see also the USP8 entry) ^37^	Disrupts proteostasis/receptor signaling by co-inhibiting OTUB1 and USP8 (UBE2N, EGFR)	[74,75,160]

^1^ *WP1130 (Degrasyn) is a non-selective DUB inhibitor that has been reported to inhibit multiple DUBs, including USP9X, USP5, USP14, and UCHL5. Accordingly, its cellular effects cannot be attributed specifically to USP9X without independent genetic or biochemical validation.* ^2^ *G9 (EOAI3402143) is a WP1130-derived analogue reported to inhibit USP9X together with USP24 and USP5. The original study indicated that complete DUB selectivity could not be fully achieved. Its cellular effects therefore cannot be attributed specifically to USP9X inhibition.* ^3^ *IU1 and its analogues are reversible USP14-directed inhibitors reported to show selectivity over other DUBs; however, the scope of counter-screening differs among studies.* ^4^ *b-AP15 and VLX1570 are structurally related α,β-unsaturated carbonyl (Michael acceptor) compounds that inhibit the proteasome-associated DUBs USP14 and UCHL5 through covalent modification and can additionally engage multiple unrelated proteins. Genome-wide and proteomic screening indicates that inhibition of USP14 and UCHL5 alone does not fully account for their cellular effects. VLX1570 also exhibited dose-limiting and ultimately fatal pulmonary toxicity during early clinical evaluation, highlighting the challenges associated with therapeutic targeting of proteasome-associated DUBs.* ^5^ *ML323 selectivity was assessed against a five-member USP panel and a 451-kinase panel; broader DUB-wide selectivity profiling has not been reported.* ^6^ *GW7647 was identified as a USP1/UAF1 inhibitor in a high-throughput screen of bioactive compounds. It is primarily a PPARα agonist and has also been reported to inhibit USP12; therefore, its cellular effects should not be attributed exclusively to USP1 inhibition.* ^7^ *UbV.10.1 is a phage display-derived ubiquitin variant rather than a small-molecule inhibitor and is included for comparison of distinct target-engagement strategies.* ^8^ *Despite its name (Specific and Potent Autophagy Inhibitor-1), spautin-1 inhibits both USP10 and USP13 and has additionally been reported to inhibit mitochondrial complex I. In the corresponding study, silencing of USP10 or USP13 did not reproduce the cellular phenotype, indicating that its effects cannot be attributed to DUB inhibition alone.* ^9^ *USP25 and USP28 are closely related homologues with nearly identical inhibitor-binding pockets, and the reported inhibitors of this subfamily engage both enzymes with comparable potency. Accordingly, their cellular effects cannot be attributed specifically to USP28 inhibition.* ^10^ *ML364 is an NIH Molecular Libraries chemical probe; the scope of its DUB selectivity and counter-screening should be interpreted according to the profiling reported in the primary study.* ^11^ *LCAHA and LCAE were profiled against 32 DUBs spanning three families and showed preferential activity toward USP family enzymes over OTU and JAMM/MPN+ proteases; however, selectivity within the USP family was not established.* ^12^ *Neutral Red is a phenazine dye widely used as a vital stain. Although the primary report described inhibition of USP4 without measurable activity against four other DUBs, its relatively weak potency (IC50 ≈ 50 μM) and cellular use at concentrations up to 200 μM preclude considering its selectivity as established.* ^13^ *For U4-I05, potential off-target activity was evaluated only against USP46 using an indirect cellular readout; broader DUB-panel profiling has not been reported.* ^14^ *USP5-IN-1 binds the zinc-finger ubiquitin-binding domain (ZnF-UBD) of USP5 rather than its catalytic site, thereby interfering with the engagement of unanchored ubiquitin chains. Its reported affinity is in the low-micromolar range, and selectivity over other DUBs or ZnF-UBD-containing proteins has not been established.* ^15^ *Compound 61 (OTUB1/USP8-IN-1) inhibits OTUB1 and USP8 with similar potency (IC50 = 0.17 and 0.28 nM, respectively) and should therefore be considered a dual inhibitor rather than an OTUB1-selective probe. Broader DUB family profiling has not been reported.* ^16^ *DC-U4106 was counter-screened against USP2 (IC50 = 58.4 μM) and USP7 (IC50 > 100 μM) only; broader DUB-panel profiling has not been reported.* ^17^ *Mitoxantrone is a clinically approved type II topoisomerase inhibitor that was subsequently reported to inhibit USP11. It is not a selective USP11 inhibitor or probe; therefore, cellular effects observed following mitoxantrone treatment cannot be attributed specifically to USP11 inhibition.* ^18^ *Despite its name (Specific and Potent Autophagy Inhibitor-1), spautin-1 inhibits both USP10 and USP13 and has additionally been reported to inhibit mitochondrial complex I. In the corresponding study, silencing of USP10 or USP13 did not reproduce the cellular phenotype, indicating that its effects cannot be attributed to DUB inhibition alone.* ^19^ *GSK2643943A is available as a research reagent with a reported USP20 IC50 in the low-nanomolar range; however, no peer-reviewed characterization establishing its DUB selectivity was identified.* ^20^ *BAY-805 is a jointly developed Bayer/SGC chemical probe supplied with a structurally matched inactive control compound (BAY-728), which allows target-dependent effects to be distinguished from compound-related artifacts. Its biochemical characterization is extensive, whereas cellular validation remains comparatively limited.* ^21^ *These flavonoids were identified through molecular docking and cellular assays measuring H2A/H2B monoubiquitination rather than through biochemical DUB assays and are active only at high micromolar concentrations. Given the broad activity often observed with flavonoids across multiple protein families, these compounds should not be regarded as selective USP22 probes.* ^22^ *Plumbagin is a naphthoquinone natural product that was reported in the primary study to inhibit DUBs, “especially USP31,” indicating activity beyond a single DUB. The accompanying specificity experiments addressed which DUB interacts with GPX4 rather than the DUB selectivity of plumbagin itself. Systematic DUB-panel profiling of the compound has not been reported.* ^23^ *Compound 39 was evaluated by activity-based protein profiling mass spectrometry against 49 endogenous DUBs spanning the USP, OTU, UCH, and Josephin families in neuronal cells, with no significant off-target DUB engagement detected.* ^24^ *MF-094 inhibits USP30 with an IC50 of approximately 120 nM and was counter-screened against a 22-member DUB panel (Ubiquigent DUBprofiler), showing less than 30% inhibition at concentrations up to 10 μM. Additional testing against USP1, USP8, and USP9 yielded IC50 values >10 μM.* ^25^ *15-Oxospira milactone is a covalent natural product that modifies the catalytic cysteine of USP30. Because covalent natural products of this type may engage cysteine-dependent enzymes, and systematic selectivity profiling has not been reported, its USP30 selectivity remains to be established.* ^26^ *IMP-1710 is a covalent inhibitor and activity-based probe for UCHL1 reported to be selective over other DUBs; the cyanopyrrolidine chemotype has also been associated with engagement of aldehyde dehydrogenases and PARK7.* ^27^ *GK13S was profiled against all four human UCH-family DUBs (UCHL1, UCHL3, UCHL5, and BAP1) and by activity-based protein profiling mass spectrometry, which identified PARK7 and C21orf33 as additional targets. It is provided as a chemogenomic pair with the inactive stereoisomer GK16S and warhead-deficient control compounds.* ^28^ *LDN-91946 was counter-screened against the closely related DUB UCH-L3 and against isopeptidase T, papain, caspase-3, and tissue transglutaminase, with no detectable inhibition. However, systematic profiling across the broader DUB family has not been reported.* ^29^ *LDN-57444 is the most widely used UCHL1 tool compound; however, direct comparison studies indicate that it does not achieve complete inhibition of cellular UCHL1, whereas GK13S does.* ^30^ *Perifosine is an alkylphospholipid Akt-pathway inhibitor; its reported activity against UCHL3 represents a repurposing observation rather than its primary pharmacological mechanism.* ^31^ *TCID inhibits UCH-L3 with an IC50 of approximately 0.6 μM and was counter-screened only against the closely related UCH-L1, against which it showed >100-fold selectivity. Broader DUB-panel profiling has not been reported.* ^32^ *Although described as specific, capzimin showed only >5-fold selectivity for RPN11 over related JAMM proteases. The reported >2-log selectivity applies to structurally unrelated metalloenzymes such as HDAC6, matrix metalloproteinases, carbonic anhydrase II, and glyoxalase 1.* ^33^ *Reduced thiolutin acts as a zinc chelator and inhibits the JAMM metalloproteases, including RPN11, CSN5, AMSH, and BRCC36.* ^34^ *SOP11 is an optimized epidithiodiketopiperazine with fewer non-specific effects than earlier analogues; however, compounds of this class also inhibit other JAMM proteases including CSN5 and AMSH.* ^35^ *DUBome-wide selectivity profiling in the primary report showed that SB1-F-70 inhibits USP30 and UCHL1 in addition to JOSD1. Therefore, cellular effects of this compound cannot be attributed specifically to JOSD1 inhibition.* ^36^ *HY041004 was counter-screened only within the Josephin (MJD) family, where it inhibited JOSD2 (IC50 = 0.26 μM) and JOSD1 (IC50 = 0.47 μM) with comparable potency and inhibited Ataxin-3 and Ataxin-3L more weakly. Profiling against USP-, OTU-, or UCH-family DUBs has not been reported; therefore, cellular effects cannot be attributed specifically to JOSD2 rather than JOSD1.* ^37^ *Compound 61 (OTUB1/USP8-IN-1) inhibits OTUB1 and USP8 with similar potency (IC50 = 0.17 and 0.28 nM, respectively) and should therefore be considered a dual inhibitor rather than an OTUB1-selective probe. Broader DUB family profiling has not been reported.*

## Data Availability

No new data were created or analyzed in this study. Data sharing is not applicable to this article.

## Abbreviations

The following abbreviations are used in this manuscript:

53BP1	p53-binding protein 1
DDR	DNA damage response
dsDNA	Double-stranded DNA
DUB	Deubiquitinating enzyme
FASN	Fatty acid synthase
GLUT4	Glucose transporter type 4
HAUSP	Herpesvirus-associated ubiquitin-specific protease
HIF-1α	Hypoxia-inducible factor 1-α
JAMM	JAB1/MPN/MOV34 metalloenzyme
K48/K63	Lysine 48/63-linked polyubiquitin chains
Mcl-1	Induced myeloid leukemia cell differentiation protein
MCPIP	Monocyte chemotactic protein-induced protein
MDM2	Mouse double minute 2 homolog
MINDY	Motif interacting with Ub-containing novel DUB family
MJD	Machado–Josephin domain
NF-κB	Nuclear factor kappa-light-chain-enhancer of activated B cells
OTU	Ovarian tumor protease
PPPDE	Permuted papain fold peptidases of dsRNA viruses and eukaryotes
PTEN	Phosphatase and tensin homolog
PSMD14	26S proteasome non-ATPase regulatory subunit 14
TGF-β	Transforming growth factor β
TGFBR1	TGF-β receptor type 1
Ub	Ubiquitin
UCH	Ubiquitin C-terminal hydrolase
UPS	Ubiquitin–proteasome system
USP	Ubiquitin-specific protease
ZUP1	Zinc finger-containing ubiquitin peptidase 1
